# First Insights into the Genome of the Amino Acid-Metabolizing Bacterium *Clostridium litorale* DSM 5388

**DOI:** 10.1128/genomeA.00754-14

**Published:** 2014-07-31

**Authors:** Anja Poehlein, Hamed S. Alghaithi, Lenin Chandran, Cynthia M. Chibani, Elena Davydova, Karthikeyan Dhamotharan, Wanwan Ge, David A. Gutierrez-Gutierrez, Advait Jagirdar, Bahar Khonsari, Kamal Prakash P. R. Nair, Rolf Daniel

**Affiliations:** a Genomic and Applied Microbiology and Göttingen Genomics Laboratory, Georg-August University, Göttingen, Germany; b Members of the Applied Bioinformatics in Microbiology Course of the Microbiology and Biochemistry MSc/PhD program, Georg-August University, Göttingen, Germany

## Abstract

*Clostridium litorale* is a Gram-positive, rod-shaped, and spore-forming bacterium, which is able to use amino acids such as glycine, sarcosine, proline, and betaine as single carbon and energy sources via Stickland reactions. The genome consists of a circular chromosome (3.41 Mb) and a circular plasmid (27 kb).

## GENOME ANNOUNCEMENT

The obligate anaerobic spore-forming bacterium *Clostridium litorale* is Gram-positive and rod-shaped. It is able to utilize amino acids such as glycine, sarcosine, proline, and betaine as sole carbon and energy sources via Stickland reactions (1, 2). *C. litorale* belongs, like its closest relative *Eubacterium acidaminophilum*, to cluster XI of clostridia (3, 4). The *C. litorale* type strain DSM 5388 was isolated from anoxic marine mud of the North Sea, Germany (1).

*C. litorale* was cultivated under anaerobic conditions using a mixture of alanine (30 mM) and betaine (50 mM) as substrate (5). The MasterPure complete DNA purification kit (Epicentre, Madison, USA) was used to isolate chromosomal DNA of *C. litorale* DSM 5388. The extracted DNA was employed to generate 454-shotgun and Illumina-shotgun libraries according to the protocols of the manufacturers. The libraries were sequenced using a 454 GS-FLX system (Titanium chemistry, Roche Life Science, Mannheim, Germany) and Genome Analyzer II (Illumina, San Diego, CA, USA). Sequencing resulted in coverages of 22.31 and 64.42, respectively. The assembly of the genome sequence was performed with the Roche Newbler assembly software 2.9, Mira 3.4, and SPAdes 3.0 (6), and resulted in 22 contigs.

The draft genome of *C. litorale* DSM 5388 comprises a single chromosome (3.41 Mb) and a circular plasmid (27 kb) with an overall G+C content of 41.3%. Automatic gene prediction was performed using the software tools YACOB and GLIMMER (7). Identification of rRNA and tRNA genes was done with RNAmmer and tRNAscan, respectively (8, 9). The IMG ER (integrated microbial genomes-expert review) system (10, 11) was used for automatic annotation, which was subsequently manually curated by using the Swiss-Prot, TREMBL, and InterPro databases (12). The draft genome harbored 14 rRNA operons, 77 tRNA genes, and 2,159 protein-encoding genes with function prediction and 933 genes coding for hypothetical proteins.

The genome of *C. litorale* harbors all genes coding for proteins necessary for degradation of glycine, sarcosine, and betaine. Two putative gene clusters encoding a glycine reductase complex were detected. In addition, one putative gene cluster for a betaine reductase complex and one for a sarcosine reductase complex were identified. These gene clusters showed a structure similar to those identified in *C. sticklandii*, *C. difficile*, *Sporomusa ovata*, or *E. acidaminophilum* (13–15). We could also identify a proline reductase gene cluster, which contains a gene coding for a protein (PrdC) with high sequence similarity to the C subunit of the Rnf complex. This gene is also present in *C. sticklandii* (13). Subunits A (GrdA) and B (GrdB, GrdF, GrdH, PrdB) of all these reductases are selenocysteine-containing proteins. In addition, we detected all genes (*selAB*C) and the tRNA necessary for incorporation of selenocysteine into proteins. We could also identify a small selenocysteine containing protein (PrpU), which harbors a CxxU redox active motif. This has been previously only identified in *E. acidaminophilum* (15).

### Nucleotide sequence accession numbers.

This whole-genome shotgun project has been deposited at DDBJ/EMBL/GenBank under the accession no. JJMM00000000. The version described here is version JJMM01000000.
